# Weight-Based Policy of Hepatitis B Vaccination in Very Low Birth Weight Infants in Taiwan: A Retrospective Cross-Sectional Study

**DOI:** 10.1371/journal.pone.0092271

**Published:** 2014-03-17

**Authors:** Chien-Yi Chen, Huey-Ling Chen, Hung-Chieh Chou, Po-Nien Tsao, Wu-Shiun Hsieh, Mei-Hwei Chang

**Affiliations:** 1 Graduate Institute of Clinical Medicine, National Taiwan University College of Medicine, Taipei, Taiwan; 2 Department of Pediatrics, National Taiwan University Hospital and National Taiwan University College of Medicine, Taipei, Taiwan; Kaohsiung Medical University Hospital, Kaohsiung Medical University, Taiwan

## Abstract

**Background:**

The current recommendation of giving the first dose of hepatitis B vaccine to very low birth weight (VLBW) infants at 30 days of chronologic age usually is not practical, because most VLBW infants are not medically stable at that age. We use an alternative body-weight-based protocol, and evaluate its efficacy in an endemic area under a universal immunization program.

**Methods:**

The immunogenicity of the current hepatitis B vaccination strategy in 155 VLBW preterm infants was evaluated at age 2 to 13 years, with parental consent. All of the infants were born between 1995 and 2006, and received their first dose of hepatitis B vaccine when they reached 2,000–2,200 g, irrespective of chronological age. Hepatitis B immunoglobulin (HBIG) was given at birth to infants born to HBsAg(+)/HBeAg(+) mothers.

**Results:**

All 155 of the recruited children were HBsAg and anti-HBc negative. The anti-HBs seropositivity rate (geometric mean titer) was 84.1% (146.5 mIU/mL) for children under 3 years, 73.5% (68.8 mIU/mL) for 4- to 7-year-olds, 27.7% (55.4 mIU/mL) for 8- to 11-year-olds and 20% (6.0 mIU/mL) for children ≥12 years of age. More than 90% of these children received the first vaccination after 30 days of age, half (51%) at 60 to 90 days, and 29 children (18.6%) after 90 days of age. Of the 26 infants born to HBsAg(+) mothers, 6/6 infants of HBeAg(+) mothers received HBIG at birth, and 12/20 infants of HBeAg(−) mothers received HBIG. None of the 26 infants became infected.

**Conclusions:**

Delaying hepatitis B vaccinations in VLBW preterm infants until they reach a weight of 2,000 g, with the administration of HBIG at birth for infants of HBsAg(+) mothers provided adequate immunogenicity and protection in a highly endemic area. Weight-based policy of hepatitis B vaccination is an effective and practical alternative strategy for VLBW infants.

## Introduction

Hepatitis B virus (HBV) infection persists as a major global health problem. More than 350 million people are chronically infected, and approximately 15 to 40% of them develop serious complications, such as cirrhosis, hepatic failure, or hepatocellular carcinoma. In endemic areas, such as Taiwan, the HBV carrier rate in the adult population reaches as high as 10–20%, and the major route of transmission is from mothers to infants. Combined administration of hepatitis B immunoglobulin (HBIG) and HBV vaccine after birth can prevent both vertical (mother-to-infant) and horizontal transmission of HBV [1]–[5], as well as childhood hepatocellular carcinoma and fulminant hepatitis[6]–[8]. Therefore, the World Health Organization and the American Academy of Pediatrics (AAP) have recommended that all neonates should receive the HBV vaccine [9]. Infant HBV vaccination strategies differ from country to country, depending on the prevalence of HBV infection and the country’s resources. In general, it is recommended that three doses of HBV vaccine, starting at birth, be integrated into routine infant vaccination programs. The use of HBIG after delivery is also recommended to further reduce the risk of HBV transmission to infants born to HBsAg-carrier mothers (as in the USA), or infants born to HBsAg/HBeAg double positive mothers (as in Taiwan) [10].

The above-mentioned vaccination program has been based on studies in term babies, and may not be suitable for very low birth weight (VLBW) infants with birth weight below 1500 g, because of their weaker immune response. Data on the efficacy and safety of HBV vaccination in VLBW infants is limited. Recommendations for VLBW infants in different countries are based either on small-scale studies or on expert opinion. Several studies have confirmed that hepatitis B surface antibody (anti-HBs) seroconversion rates are not satisfactory in preterm infants when the vaccine is given at birth or before the infant reaches a weight of 2,000 g [11]–[14]. Therefore, an AAP policy statement published in 1994 recommended delaying the first dose of hepatitis B vaccine in infants weighing less than 2,000 g who are born to HBsAg-negative mothers until those infants have reached 2,000 g or 2 months of age [15]. Two subsequent case studies reported in 1997 demonstrated that 90 to 95% of preterm infants with birth weight less than 2,000 g seroconverted in response to hepatitis B vaccines started at 1 month of age [16] or before discharge [17], and their mean body weights were around 2,200–2,300 g when they received the first dose of vaccine. Another study performed in 25 healthy VLBW infants reported a 96% response rate at age 6 months when the first dose of hepatitis B vaccine was given at 1 month of age. [14] Therefore, an updated AAP recommendation in 2003 suggested that medically stable infants with birth weights less than 2,000 g who were born to HBsAg-negative mothers should receive the first dose of hepatitis B vaccine as early as 30 days of chronologic age, regardless of gestational age or birth weight [18]. For infants who were born to carrier mothers and who weighed less than 2,000 g, the AAP recommends that the birth dose of hepatits B vaccine should be given with HBIG for immunoprophylaxis, but should not have counted as part of the 3 doses of the HBV immunization series.

However, these recommendations are not clinically practical for VLBW preterm infants, because most of them are not medically stable or have not achieved steady weight gain by 30 days of chronologic age. This raises concerns regarding safety and immunogenicity related to early vaccination. For the preterm infants who are still medically unstable at 1 month of age, the timing of the first dose of hepatitis B vaccine is not established, and may be missed or delayed, which may cause practical problems for clinical staff. Furthermore, there are no follow-up data to support long-term protection of VLBW infants receiving HBV vaccinations at a time when body weight is inadequate. Because there have been increasing numbers of VLBW infants in recent years, evidence-based data are required for optimization of the immunization schedule for VLBW infants.

A universal hepatitis B vaccination program was launched in July 1984 in Taiwan, one of the earliest in the world. In this program, all pregnant women are screened for serum HBsAg and HBeAg prenatally, and all infants receive three doses of HBV vaccine, at birth and at 1 and 6 months of age. In addition, infants of HBsAg- and HBeAg-positive mothers receive 0.5 ml of HBIG within 24 hours of birth. The current hepatitis B vaccination policy for preterm infants is weight-based, as per the earlier recommendation of the AAP [15]. All preterm infants follow the same hepatitis B vaccination and HBIG schedules as term infants, except that the first dose is delayed until a weight of 2,000–2,200 g has been reached, regardless of chronological age. This policy is practical and easy to follow for clinical practitioners; however it raised concerns that delayed vaccination may increase the risk of HBV transmission in VLBW infants, especially in endemic areas such as Taiwan. VLBW infants also were at greater risk of horizontal transmission of infection in early infancy due to prolonged stay in the hospital and in the intensive care unit. Despite the success of universal immunization in decreasing the HBsAg carrier rate from >10% to <1% in term infants in Taiwan [19], there have been no clear data on the rate of HBV infection in premature infants. To address this important issue in this special group of children, we conducted the present study to evaluate the efficacy and immunogenicity of hepatitis B vaccinations administered according to our weight-based policy of VLBW preterm infants.

## Methods

### Study Population

This is a retrospective cross-sectional study approved by Institutional Review Board of the National Taiwan University Hospital. A total of 630 surviving preterm infants with birth weights <1,500 g who were admitted to National Taiwan University Hospital between 1995 and 2006 were eligible for inclusion in this study. Six hundred and one children were invited to join the study by mail, and 29 infants were excluded due to invalid addresses. All of the subjects whose parents responded were recruited in 2008, when their chronological age was >2 years. After informed-consent forms were signed by the parents of the enrolled children, 3–8 mL of blood was obtained from each child via venipuncture. The children’s immunization records, including the dates of each vaccine and HBIG, were confirmed by hospital charts and by immunization records in the official child health booklets kept by the parents. The following information was obtained from medical chart review at enrollment: gestational age, birth weight, birth length, birth head circumference, gender, delivery mode, Apgar score, HBIG administration, and length of neonatal care unit and hospital stay. The results are expressed as the mean±SD or numbers of infants and percentage of the total.

### Vaccination Program

All healthy, full-term newborns received three doses of recombinant HBV vaccine, Engerix-B (20 μg/1 mL; SmithKline Beecham, Rixensart, Belgium) or H-B-Vax II (5 μg/0.5 mL; Merck Sharp & Dohme, Rahway, NJ, USA) at first day, 1 month, and 6 months of age. In addition, HBIG 0.5 mL (100 IU) was given within 24 hours after birth to newborns of HBsAg-carrier mothers with positive HBeAg or high HBsAg titers (reciprocal titer >1∶2560 by reverse passive hemagglutination test). The hepatitis B vaccination policy for preterm infants is nearly the same as for full-term newborns, except for the delay of the first dose. Before Jan 2005, the first dose was administered once the infant’s body weight exceeded 2,200 g. The body-weight requirement for the first vaccination was adjusted to 2,000 g in Jan 2005. Two subsequent doses are administered 1 month and 6 months after the first vaccination. Because of preterm delivery (prior to 32 weeks), not all mothers of VLBW infants had been screened for HBsAg and HBeAg. If the maternal HBsAg carrier status was unknown within 24 hours after delivery, the staff recommended HBIG administration, but the final decision was left to the parents.

### HBV Serology

Serum HBsAg and its antibody (anti-HBs) and hepatitis B core antibody (anti-HBc) were tested in all subjects. All HBV serologic markers were determined by enzyme immunoassay (Abbott Laboratories, North Chicago, IL). Anti-HBs is considered seropositive if the titer is >10 mIU/mL. The results were presented as the rate of seropositivity or geometric mean titer with 95% confidence interval.

### Statistical Analysis

The data was expressed as the mean±standard deviation (SD) or number of infants with percentage.

## Results

**Figure 1:**
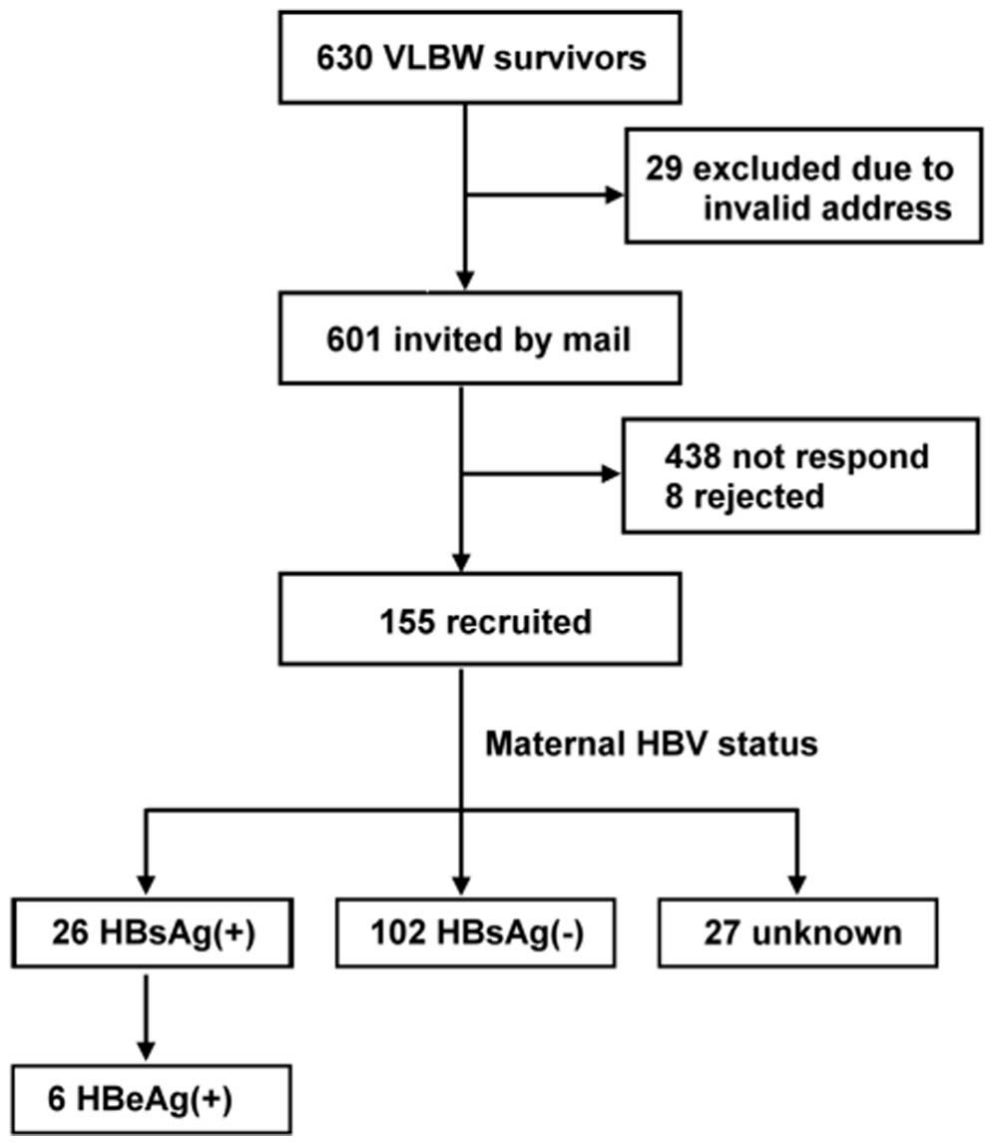
Enrollment of the study population.

A total of 155 children, who were born between 1995 and 2006 and aged 2–13 years in 2008, were recruited with parental consent (Figure 1). The total response rate was approximately 25.8%. The participants were divided into four age groups, based on their chronological age at the time of the study: <3 years, 4–7 years, 8–11 years and >12 years of age. Table 1 shows the clinical characteristics of the study population. Their mean gestational age was 29.3±2.83 weeks, and their mean birth weight was 1,144.8±268.5 g. There were 92 boys and 63 girls, and 78.1% were delivered by Cesarean section. On average, they stayed in the NICU for 45.9 days and in the hospital for 70.6 days.

**Table 1:** Clinical characteristics of the study population.

Characteristics	<3 y	4–7 y	8–11 y	>12 y
	(n = 44)	(n = 49)	(n = 47)	(n = 15)
Response rate	25.1%	24.7%	27.5%	26.3%
Gestational age (wk)	29.1±2.9	28.9±2.8	29.7±2.9	29.7±2.3
Birth weight (g)	1192.5±284	1121.9±266	1147.7±265	1071.1±251
Birth length (cm)	37.4±3.4	36.6±3.1	36.3±3.6	36.4±3.1
Birth head girth (cm)	26.7±2.3	26.3±2.0	26.4±2.4	25. 3±2.0
Number male (%)	26(59%)	26(53%)	30(64%)	10(67%)
Number C-section (%)	37(84%)	35(71%)	35(74%)	14(93%)
1-min Apgar score	4.8±2.1	5.1±1.8	4.7±2.1	4.5±2.7
5-min Apgar score	7.4±1.3	7.4±1.3	7.5±1.4	7.0±2.3
Stay in NICU (d)	49.2±35.8	45.7±23.8	35.4±19.7	63.2±26.8
Stay in hospital (d)	69.8±37.6	66.6±28.6	66.5±33.2	95.4±39.8
Age of HBV vaccine (d)				
1st dose^a^	55.2±27.2	71.6±27.8	65.2±27.1	73.9±25.3
2nd dose	96.2±29.7	106.0±31.8	101.0±28.2	108.5±27.2
3rd dose	236.2±34.7	288.9±160.5	310.0±269.1	277.1±49.0

Data are presented as mean±SD or numbers of infants (%). The age was the birth year.

a In those <3 years, i.e. born after Jan. 2005, the body weight at first HBV vaccination was >2000 g. For those born before 2005, the first dose was given at body weight >2,200 g.

### Age at First HBV Vaccination

The HBV vaccination schedule was delayed, based-on weight criteria in our study population. Because of the change of the government policy in 2005, all the children in the <3 years group (n = 44) received the first dose of HBV vaccination when their body weights exceeded 2,000 g. The other children in this study (n = 111) had received their first HBV vaccination when the body weight exceeded 2,200 g. The different policy made the children in the <3 years group received earlier vaccination program than the other age groups >3 years old (older age group, table 1 ). Overall, the mean ages at first, second, and third dose of HBV vaccination were 65.2, 101.9, and 278.4 days, respectively. A total of 94% of the study population (146 of 155 children) had their first dose of HBV vaccine after 30 days of age; 51% (79 of 155 children) received their first vaccination after 60 days of age; and 19% (29 of 155 children) after 90 days of age.

### Maternal HBV Carrier Status

Twenty-six children among 128 children who had maternal HBV data at delivery were born to HBsAg-positive mothers. Six of the 26 mothers were also HBeAg-positive (Table 2). All six children born to HBsAg-positive, HBeAg-positive mothers received HBIG within 24 hours. Their mean age at first HBV vaccination was 57.8 days, ranging from 26 to 83 days. Of the 20 children who were born to HBsAg-positive, HBeAg-negative mothers, eight did not receive HBIG after birth by our immunization program. The first doses of HBV vaccine in these eight children were all delayed beyond 60 days of age (mean 81.5 days), except in one case, who received the first dose at 34 days of age. Twenty of the 26 infants born to carrier mothers were fed only with their mothers’ breast milk. None of these children became infected with HBV.

**Table 2:** Maternal hepatitis B carrier status of the study population.

	<3 y	4–7 y	8–11 y	>12 y	Total
	(n = 44)	(n = 49)	(n = 47)	(n = 15)	(n = 155)
Maternal status at study					
Known	36	40	38	14	128(82.6%)^a^
Unknown	8	9	9	1	27(17.4%) a
Maternal HBsAg (+)	5	10	7	4	26(16.8%) a
HBsAg(+)/HBeAg (+)^b^	2	2	0	2	6
HBsAg(+)/HBeAg (−)	3	8	7	2	20
HBIG at birth^a^	11	10	6	4	31(20%) a
Unknown maternal status at delivery	6	3	2	2	13
Maternal HBsAg(+)/HBeAg(+)	2	2	0	2	6
Maternal HBsAg(+)/HBeAg(−)	3	5	4	0	12
Maternal status at study	36	40	38	14	128(82.6%)^a^

a The percentage was all calculated by case number divided with total number (n = 155).

b Maternal HBsAg (+): maternal positive HBsAg tested during pregnancy; Maternal HBeAg (+): maternal positive HBeAg tested during pregnancy;

Twenty-seven children had unknown maternal HBV status at the time of delivery because they were born before the schedule of maternal HBsAg/HBeAg screening. HBIG was administered to 18 of 27 children. None of these children was positive for HBsAg or anti-HBc.

### HBV Serology

None of the 155 children was seropositive for HBsAg or anti-HBc, indicating that no transmission of HBV infection was identified in the study population (Table 3) either vertically (perinatally) or horizontally. The geometric mean concentration of anti-HBs decreased as age increased. The anti-HBs seropositivity rate was 84.1% (73.28%–94.9%) in children 3 years of age and younger, 73.5% (61.1%–85.8%) at 4–7 years, 27.7% (14.9%–40.5%) at 8–11 years, and 20% (0%–40.2%) after 12 years of age. Although the decrease in response rate was more significant after 8 years of age, the seropositivity of HBsAg and anti-HBc was not increased. Horizontal transmission of infection was not significant.

**Table 3:** The results of hepatitis B virus serology of the study population.

	<3 y	4–7 y	8–11 y	>12 y
	(n = 44)	(n = 49)	(n = 47)	(n = 15)
HBsAg seropositivity	0%	0%	0%	0%
Anti-HBc seropositivity	0%	0%	0%	0%
Anti-HBs seropositivity^a^	84.1%	73.5%	27.7%	20%
(95%CI)	(73.3–94.9)	(61.1–85.8)	(14.9–40.5)	(0–40.2)
Anti-HBs GMT (mIU/ml)	146.5	68.8	55.4	6.0
(95%CI)	(80.4–212.5)	(34.8–102.7)	(3.1–107.7)	(0.03–12.0)

a Anti-HBs seropositivity: defined as serum level >10 mIU/ml.

GMT: geometric mean titer.

## Discussion

In the present study, conducted in an HBV-endemic area, no HBV infection was identified in 155 VLBW children under a weight-based vaccination program. The low prevalence of HBsAg (0%) is comparable to that of children born at term after universal vaccination began in Taiwan (0.9%), and is much lower than the rate among children born before the vaccination program began (10%) [20]. Although the children in our study received their first dose of vaccine at a mean age >55 days, with the majority (94%) starting the first dose after 30 days, which is much later than the current AAP recommendation, none of the children was seropositive for HBsAg or anti-HBc. The maternal HBV carrier rate in the present preterm population was approximately 16.8%, which is in accordance with that of the general population [19]. For the VLBW infants born to HBV-carrier mothers, HBIG was used for passive immunization immediately after birth, especially in infants with HBsAg and HBeAg double-positive mothers. Although we did not use the birth dose of hepatitis B vaccine as part of immunoprophylaxis in infants born to carrier mothers during study periods, none of the 26 infants was infected. This observation demonstrates that our weight-based policy of hepatitis B vaccination in preterm infants, in comparison to the age-based policy, did not increase the risk of HBV infection in an endemic area.

According to AAP recommendations, early initiation of HBV immunizations at 1 month of chronologic age provides timely protection to vulnerable preterm infants, who are more likely than other children to receive transfusions or surgical interventions. The theoretical risk of horizontal transmission of infection from health-care providers, family members, and caretakers also would be minimized; however, the weight-based policy in the present study did not actually increase the risk of HBV infection. In addition to the protective effect of HBIG at birth, advances in NICU care for preterm infants may be important factors. For example, infection control measures, such as hand washing, aseptic technique, and limited visitors during NICU hospitalization may minimize HBV exposure and decrease the risk of infection in preterm infants. Of note is that there has been no restriction of HBV-carrier health-care workers in the NICU in Taiwan. Advanced screening of donor blood before transfusion also contributes to the low infection rate. Furthermore, some VLBW infants, especially those who are very preterm, are not medically stable at 1 month of chronologic age, and are not suitable for vaccination. The timing to initiate hepatitis B vaccination in these cases is uncertain, and the first dose may then be missed or more delayed. Delaying hepatitis B vaccinations in VLBW preterm infants until they reach a weight of 2,000 g is a practical strategy for clinical health providers, because by the time preterm infants reach this goal, most are medically stable, have achieved adequate muscle mass, and are suitable for vaccination.

Another important finding in the present study is the high anti-HBs seroprotection rate in preterm infants under the weight-based vaccination policy. The major concern of early HBV vaccination in preterm infants is the lower rate of seroprotection and lower rate of antibody persistence. In one Alaskan study with an age-based policy, 37 preterm infants had their first HBV vaccination at a mean age of 7 days, and their seroconversion rate was only 8.1% at 3 years of age [21]. In our study, the mean age of first vaccination was 65.2 days, and the anti-HBs response rate was 84.1% at 2–3 years of age. This rate is satisfactory, and is comparable to the rates for term infants; the anti-HBs seropositivity rate in a Taiwanese vaccinated birth cohort was 80.8% at 3–4 years of age [19]. Similarly, Linder et al. [22] also report a high rate of seroprotection in preterm infants whose first HBV vaccination was delayed until they weighed 2,000 g. This observation is not unexpected because poor weight gain in the first 6 months of life is associated with an inadequate immune response in premature infants. [13] Most VLBW infants could mount a better immune response if they have reached a weight of 2,000 g, at which point they may already show steady weight gain. Our study further addressed the benefits of delayed vaccination in preterm infants. It is possible that the relatively higher dose of HBV vaccine, i.e. 20 ug of HBV vaccine per dose, given to our infants in routine HBV immunization may contribute to the relatively higher immunogenicity in our infants.

Although early HBV immunogenicity in premature infants has been studied, the data for long-term efficacy is limited. Only one study evaluated the immune response of 16 former extremely preterm infants at 7 years of age, and the reported anti-HBs seropositive rate was 86% [23]. In the present study, we assessed the antibody response to hepatitis B vaccinations in children up to 13 years of age who had very low birth weights. The anti-HBs response rate in our study was as high as 73.5% in children up to 7 years of age. This rate is comparable to data from the vaccinated birth cohorts in the general population [19]; however, a rapid decrease in the seropositivity rate, to 20–27%, was significant in our study population after 8 years of age. The persistence rate of anti-HBs is much higher in the birth cohort of the vaccinated general population: 43.3% at 9–10 years and 41.7% at 11–12 years. This difference may be caused by multiple factors influenced by prematurity. Most studies reported lower seroconversion rates in preterm infants than in term infants [21], [23], [24], but the cause of this is unclear. Alterations in immune responses caused by preterm birth may be involved. Further investigation is warranted to clarify the influence of prematurity on long-term immunogenicity.

Universal screening of pregnant women for hepatitis B and passive immunization with HBIG are highly effective measures for prevention of HBV in children born to carrier mothers [10]. It is very important that preterm infants born to HBV-carrier mothers receive timely protection by HBIG before active immunity can be achieved by HBV vaccination, in those with HBV-carrier mothers or those with maternal unknown carrier status. In Taiwan, all pregnant women have routine HBV screening with HBsAg and HBeAg at approximately 32 weeks of gestation; however, some mothers deliver prematurely, before their scheduled HBV screening, as was the case with the 27 mothers with unknown HBV status in our present study. This is a common practical issue when caring for preterm infants, and may underestimate the prevalence of carrier mothers. Some preterm infants may not receive HBIG, and thus have an increased risk of infection if their first vaccination is delayed. Therefore, we advocate the official schedule time of maternal HBV screening before 24 weeks of gestation, for better care of infants born prematurely. It is not easy to enroll large number of extremely premature infant to follow up their HBV markers; however, continuous efforts to accumulate more cases of VLBW infants after different strategies may help to define the best strategy for HBV immunization in VLBW infants.

## Conclusions

Delaying hepatitis B vaccination in VLBW preterm infants until they weigh 2,000 g, a weight-based policy, provides adequate immunogenicity in an HBV endemic area. Adequate long-term seropositivity rates and geometric mean titers of anti-HBs in these infants were comparable to those of term infants. No evidence of horizontal transmission of infection was noted up to the age of 13 years. In addition, for VLBW infants born to mothers who are seropositive for both HBeAg and HBsAg, HBIG should be given to the infants immediately after birth for early protection prior to the HBV vaccination.
